# Serological, cultural and molecular evidence of *Brucella melitensis* infection in goats in Al Jabal Al Akhdar, Sultanate of Oman

**DOI:** 10.1002/vms3.103

**Published:** 2018-05-23

**Authors:** Yasmin ElTahir, Al Ghalya Al Toobi, Waleed Al‐Marzooqi, Osman Mahgoub, Maryne Jay, Yannick Corde, Hadi Al Lawati, Shekar Bose, Abeer Al Hamrashdi, Kaadhia Al Kharousi, Nasseb Al‐Saqri, Rudaina Al Busaidi, Eugene H. Johnson

**Affiliations:** ^1^ College of Agricultural & Marine Sciences Department of Animal & Veterinary Sciences Sultan Qaboos University Alkhod Sultanate of Oman; ^2^ EU/OIE/FAO & National Reference Laboratory for Brucellosis Animal Health Laboratory Paris‐Est University/Anses Maisons‐Alfort France; ^3^ Ministry of Agriculture & Fisheries Directorate General of Animal wealth Muscat Sultanate of Oman; ^4^ Department of Natural Resources Economics Sultan Qaboos University Alkhod Sultanate of Oman

**Keywords:** Brucellosis, *Brucella*, Al Jabal Al Akhdar, Oman

## Abstract

Brucellosis, one of the most common zoonotic diseases and has significant public health and economic importance worldwide. Few studies and reports have been performed to estimate the true prevalence of animal brucellosis in the Sultanate of Oman; however, no incidence of the disease was previously reported in Al Jabal Al Akhdar. The purpose of this study was to investigate the prevalence of brucellosis in goats in eight villages in Al Jebal Al Akhdar, Sultanate of Oman, namely: Al Aqaieb, Al Helailat, Al Ghilayil, Hail Al Hedap, Da'an Al Hamra, Shnoot, Al Qasha'e and Al Sarah**, **Al Jabal Al Akhdar in the Sultanate of Oman. In this study we used different diagnostic serological tests, namely, RBT, I‐ELISA and CFT to study the prevalence of Brucella infection in goats in Al Jabal Al Akhdar. Statistical analysis using Kappa statistics was used to compare the performance of the serological tests. Biochemical tests and species‐specific Multiplex PCR were used to identify the *brucella* species involved in the infection. A structured questionnaire and Chi‐square (x^2^) statistical analysis was used to identify related brucellosis risk factors. This study is the first to reveal brucellosis infection in goats in eight villages in Al Jebal Al Akhdar, Sultanate of Oman, namely: Al Aqaieb, Al Helailat, Al Ghilayil, Hail Al Hedap, Da'an Al Hamra, Shnoot, Al Qasha'e and Al Sarah**,** with an overall seroprevalence of 11.1%. The study also compared the performance of three different serological tests, namely, RBT, I‐ELISA and CFT. Statistical analysis using Kappa statistics showed that the degree of agreement was best seen between RBT and CFT (96%), followed by RBT, I‐ ELISA (91.4%) and CFT and I‐ ELISA (89.2%). Biochemical tests and species‐specific Multiplex PCR showed the typical profile for* B. melitensis*. A structured questionnaire and Chi‐square (x^2^) statistical analysis indicated that the presence of abortion is the major risk factor for the prevalence of brucellosis, whereas age and sex were not significant factors in the tested animals. Besides, poor knowledge about brucellosis, consumption of unpasteurized milk or milk products, free trade of animals and the introduction of new animal breeds to herds were all contributing risk factors to the prevalence of brucellosis. The prevalence of human brucellosis obtained verbally from pastoralists gave an insight that brucellosis could pose a public health hazard, especially in those high‐risk groups, mainly the pastoralists in the study area. Because of their constant and increasing interaction with their animals, pastoralists could be at a high risk of occupational infection.

## Abstract 2

Impacts:

- This study is the first to reveal that brucellosis is prevalent in goats in Al Jabal Al Akhdar, Sultanate of Oman with an overall prevalence of 11.1%.
- Statistical analysis using Kappa statistics showed the degree of agreement between the performances of three different serological tests was best seen between RBT and CFT (96%) followed by RBT and I‐ ELISA 91.4%, and CFT and I‐ ELISA 89.2%.
- The use of Bruce‐ladder multiplex PCR allowed us to detect, for the first time, *B. melitensis* in goats in Al Jabal Al Akhdar.

## Introduction

Brucellosis, one of the most common zoonotic diseases, has significant public health and economic importance worldwide (Corbel 1997; Almuneef *et al*. 2004). The disease aetiology involves bacteria of the genus *Brucella*, which are Gram‐negative, non‐motile, facultative anaerobic intracellular coccobacilli (Alton & Forsyth 1996). A wide range of mammals are targets for brucellosis, including man, cattle, sheep, goats, camels, swine and wild life (Cutler *et al*. 2005). The genus *Brucella* comprises a number of species based on pathogenicity and host preference. These include *B. abortus* (cattle), *B. canis* (dogs), *B. ovis* (sheep), *B. melitensis* (sheep and goats), *B. suis* (pigs, reindeer and hares) and *B. neotomae* (desert wood rats). Brucellosis is widely distributed in Africa, Latin America, Mediterranean, Middle East and parts of Asia that represent the endemic areas for the disease (Corbel 2006).

In the Sultanate of Oman, the disease is particularly prevalent in people in Dhofar where it was first reported in 1979 (Al‐Rawahi 2015). A study conducted by the Ministry of Health between 1995 and 2012 reported 2737 cases, with high incidence of the disease in children (0–10 years). Serological evidence of exposure to *Brucella* was reported in 1% of healthy residents of Dhofar, mainly in children (Scrimgeour *et al*. 1999), and a similar study in Dhofar showed the significance of transmission through ingestion of raw milk and contact with animals (El‐Amin *et al*. 2001). Further reports showed the presence of brucellosis in the northern as well as the southern parts of the Sultanate (Ministry of Health, 2002).

The seroprevalence of animal brucellosis reported earlier in cattle, goats, sheep and camels in Oman (Ismaily *et al*. 1988) was higher than has been recently reported for the same species (Al‐Rawahi 2015).

*Brucella melitensis* biovar 1 was the only type that was isolated from cattle, camels, sheep and goats in the southern part of the Sultanate (Adam & El‐Rashied 2013).

As for goat breed, three types were described in Oman, and these are Dhofari, Batina and Al Jabal Al Akhdar breeds (Zaibet *et al*. 2004); among these, Jabal Akhdar breed is the largest in size and the dominant species in Jabal Akhdar where livestock rearing remains a significant part of livelihood derived from meat and milk production.

Geographically, Al Jabal Al Akhdar ‘the Green Mountain’ is part of the Al Hajar Mountains range in Al Dakhiliya governorate in the northern part of the Sultanate. The area of the study is characterized by its temperate climate, being situated at an elevation between 1000 and 3000 metres above sea level where the temperature often drops below −5°C during December to March. Moreover, Sultan Qaboos designated Al Jabal Al Akhdar a nature reserve in a bid to conserve its unique yet fragile biodiversity, and a decree issued by the Royal Court established the ‘Al Jabal Al Akhdar Sanctuary for Natural Sceneries’. Regarding husbandry, goats and sheep are allowed to browse during the day, whereas cattle are always kept in barns and stall‐fed with hay made from cultivated grass and concentrates.

Few studies and reports have been performed to estimate the true prevalence of animal brucellosis in the Sultanate; however, no incidence of the disease was previously reported in Al Jabal Al Akhdar. Therefore, the purpose of this study was to investigate the prevalence of brucellosis in goats in Al Jabal Al Akhdar using different diagnostic techniques and identifying related risk factors.

## Materials and methods

### Study areas

This study was conducted at Al Jabal Al Akhdar, Sultanate of Oman. The selection of study areas shown in the map was based on the following criteria:

1. Previous pilot study indicating the presence of susceptible domestic animal hosts
2. Previous evidence of *Brucella *in the human population.

### Study design and sample size estimation

Considering the resource constraints, eight villages namely: Al Aqaieb, Al Helailat, Al Ghilayil, Hail Al Hedap, Da'an Al Hamra, Shnoot, Al Qasha'e and Al Sarah, were selected randomly based on the data on animal population obtained from the Ministry of Agriculture and Fisheries (2014). A proportional allocation method was used to determine the sample size from each randomly selected village based on the goat population numbers in each village (Table 1). A total sample size of 324 animals (*N* = 324) was determined in which the individual animals were randomly selected from both sexes at different reproductive age. For calculating prevalence, a confidence limit of 95% was set, with the proportion of attributes (p) = 0.7 (based on the pilot study results).

**Table 1:** Sample size plan used in the study

Village	Total population (heads)	% of total population	No. of individual animals to be sampled	Adjusted No. of animals sampled
Al Aqaieb	435	15.8	50.2	50
Al Helailat	508	18.6	59.8	60
Al Ghilayil	187	6.8	22.0	22
Da'an Al Hamra	343	12.5	40.4	41
Hail Al Hedap	155	5.7	18.3	18
Al Sarah	185	6.8	21.8	22
Al Qashaa	265	9.7	31.2	31
Shnoot	660	24.1	77.8	80
Total	2738	100%	321.5	324

Samples were collected in March 2015, and the health status, age, sex and history of abortion for each goat were documented, with no record of previous history of vaccination in Al Jabal Al Akhdar. All the animals sampled were maintained under a mixed management system where different species were kept together.

### Blood and milk samples collection

Blood samples were collected from the jugular vein of goats in two sets of sterile 5 mL vacutainers, one set with anticoagulant for DNA extraction and the other without anticoagulant for serum separation, all samples being kept on ice for transport to the laboratory. Sera were separated by centrifugation at 959 g for 5 min and after heat‐inactivation at 56°C for 30 min kept at −20°C before serological tests. Blood samples for DNA extraction were kept at +4°C for molecular tests.

Milk was collected from the same lactating goats that were sampled for blood. Milk sampling was done by hand stripping just prior to milking using sterile screw caped 50 mL Falcon tubes (Kartell S.p.A and Cellstar tubes, Germany). Each sample was composed of a representative amount of milk from each teat. Different volumes were taken from each udder, with the first strips being discarded. Samples were then divided into two aliquots, one for bacteriological isolation and the other for DNA extraction, and these were dispatched to the laboratory for storage at −20°C till further testing.

### Detection of *Brucella* antibodies by serological methods

All serum samples were tested in the brucellosis reference laboratory in Paris, France, according to the standards of the World Organization for Animal Health for diagnosis of brucellosis in small ruminants by using the Rose Bengal test (RBT), the complement fixation test (CFT) and the indirect enzyme‐linked immunosorbent assay (I‐ELISA) using IDEXX kits (*World Organization for Animal Health Paris: OIE, 2009)*.

### Rose Bengal test

All serum samples were screened by the RBT for the presence of antibodies against *Brucella* antigens. All samples, controls and the RBT antigen were equilibrated at room temperature prior to the test. Briefly, equal volumes of 25 *μ*L of three replicates of the positive, negative, the test sera and the RBT antigen (IDEXX, France) were placed on each side of an agglutination plate consisting of 48 white tiles. The sera and antigen were then mixed thoroughly all at once using 48‐projections plate to produce a circular zone approximately 2 cm in diameter. Then, the mixture was agitated gently for 4 min on a three‐directional agitator (Jean Robin, France), and the degree of agglutination was then recorded on a matching sheet.

### Indirect enzyme‐linked immunosorbent assay

I‐ELISA was performed using a diagnostic kit [IDEXX, France (4067)] as per the manufacturer's protocole. Briefly, after equilibration at room temperature, each sample was tested in triplicate. A volume of 190 *μ*L of the dilution buffer was added to each well followed by addition of 10 *μ*L of the controls and test sera to the dilution buffer with gentle shaking of the plate followed by incubation for 45 min (±5) at 18–26°C. The plate was then washed three times using 300 *μ*L wash buffer (1X) and dried completely. After addition of the diluted conjugate, incubation, washing and stop reaction, the optical density (OD) was measured at 450 nm using ELISA reader Multiscan GO (Thermo Fisher Scientific, USA). The positive and negative cut off was determined as 120% and 110%, respectively. The S:P ratio (sample to positive ratio) of the test samples was calculated of the mean OD of the sample, two positive and negative control wells using the following formula: S/P(%) = 100 × , NC: negative control PC: positive control.

Validation of I‐ELISA test was carried out with positive and negative controls as per the manufacturer's instruction. All samples were tested in triplicates and where the plate validation failed, the procedure was repeated. A test sample giving a S:P ratio equal to or greater than 120% was regarded as positive and the ones giving an S:P ratio less than or equal to 110% was regarded as negative. Samples with S:P percentage greater than 110% and less than 120% were considered suspect and they were retested.

### Complement fixation test

The CFT was carried out according to the World Organization for Animal Health prescribed procedure as previously described (Alton & Paris‐Grignon 1988; OIE. 2004). The test and control sera were first inactivated for 30 min at 59°C. For the test, *Brucella* antigen B115 was used, with complement diluted at 1:40. Positive and negative control sera were run on the same test plate in addition to antigen control, complement control and sensitized SRBCs control. The reaction was observed and the end point was validated by observing complete haemolysis in the control wells. The positive control serum is visualized at the expected titre ± one dilution.

### Isolation of *Brucella* from milk and blood samples

To detect the presence of *Brucella,* about 8–10 mL milk or whole blood samples from seropositive animals were inoculated into BACTEC bottles (Lytic/10 aerobic/F culture vials) and incubated for about 7 days in a BACTEC 9240 system (Becton Dickinson, USA). When a positive bottle was detected, a Gram stain for the broth was performed, and a portion of the fluid was subcultured onto 5% sheep blood agar and incubated for 3–4 days at 37°C. Bottles with negative growth index were kept for three more weeks. Cultures were considered negative if no *Brucella* was detected during the fourth week of incubation.

### Identification *of Brucella* species from *Brucella* isolates

Presumptive identification of *Brucella melitensis* or *B. abortus* was performed on the basis of the typical microscopic picture showing small Gram‐negative coccobacilli, positive for oxidase, catalase and urease tests, further confirmed by a positive agglutination with specific antisera (Remel Europe Ltd). Further identification was performed using Vitek 2 systems (version 07. 01, BioMeriux) with a Gram‐negative bacteria colorimetric identification card (GN card) that contains different biochemical tests.

### *Brucella* genomic DNA extraction from the whole blood

DNA isolation kit (Qiagen, Germany) was used to extract DNA from whole blood. The spin column protocol was followed. Briefly, a volume of 50–100 *μ*L anticoagulated blood samples was added to each collection microcentrifuge tubes followed by the addition of 20 *μ*L proteinase K, and the total volume was adjusted to 220 *μ*L with PBS. Four microlitres of RNase A (100 mg/mL) was then added to each tube and incubated for 5 min at room temperature. A volume of 200 *μ*L lysisbuffer (AL) was then added and the tubes were properly sealed using the caps provided, shaken vigorously for 15 s and then centrifuged at 5009 g for 1 min. The tubes were then incubated at 56°C for 10 min with continuous mixing. Then a volume of 200 *μ*L ethanol (96–100%) was added and the tubes were shaken vigorously for 15 s followed by centrifugation at 8000 rpm for 1 min. The mixture (maximum 900 *μ*L) was then carefully transferred to the DNeasy columns. The tubes were then centrifuged for 1 min at 8000 rpm to allow passage of the lysate through the membrane of the DNeasy columns. To remove the residual contaminants, 500 *μ*L of wash buffer AW1 was added to each sample and centrifuged for 1 min at 8000 rpm. Then, 500 *μ*L of the second wash buffer AW2 was added to each sample and centrifuged for 3 min at 15339 g. To elute the DNA, the columns were placed in an elution tubes and a volume of 100 *μ*L AE elution buffer was added to each tube and incubated for 5 min at room temperature (15–25°C). Then, tubes were centrifuged for 1 min at 8000 rpm and the eluted DNA was collected and stored at −20°C for further molecular analysis.

For DNA extraction from frozen blood samples, these were thawed and washed with the TE buffer (10 mmol/L Tris–HCl, 1 mmol/L EDTA) several times to ensure removal of the RBCs that would hinder the extraction procedure.

### *Brucella* genomic DNA extraction from milk samples

Bacterial DNA from the milk samples was extracted using a Norgen Milk Bacterial DNA Isolation Kit (Norgen biotek corp., Canada). Milk samples in microcentrifuge tubes were centrifuged at 14 000 rpm (~20 000*g*) for 3 min to obtain pellets, which were then isolated by removing the supernatant and the cream. Then, 400 *μL* of the lysis Buffer (SK) was added to each tube and mixed well by vortexing. A volume of 200 *μL* of 96–100% ethanol was then added to the lysis with vortexing. Then, the mixture was transferred to a silica‐based spin column and centrifuged for 2 min at 14 000 rpm (~20 000*g*). About 500 *μL* of Buffer SK was then added to the column and centrifuged for 2 min at 14 000 rpm (~20 000*g*). The flow‐through was discarded and the column was reassembled with the collection tube. An amount of 500 *μL* of wash solution A was then added to the column in successive column washing steps with centrifugation for 1 min at 14 000 rpm (~20 000*g*) and the flow‐through was discarded, after which the column was dried by centrifugation for 2 min at 14 000 rpm (~20 000*g*). DNA was finally eluted with 100 *μ*L of elution buffer by two successive centrifugations at 313 g (~425*g*) for 2 min. The concentration and the purity of the DNA were determined spectrophotometrically by the ratio of the *A*_260_ and *A*_280_ values using Nano drop 2000 (Thermo Fisher Scientific, USA).

### *Brucella* genomic DNA extraction from bacterial isolates

To extract DNA from bacterial isolates, 3–4 bacterial colonies were picked up from the blood agar and added to 1.5 mL microcentrifuge tube containing 200 *μ*L PBS. The tubes were vortexed to dissolve the colonies and about 20 *μ*L proteinase K was added. A volume of 4 *μ*L RNase A (100 mg/mL) was then added to each tube and incubated for 5 min at room temperature. Then, a volume of 200 *μ*L lysis buffer (AL) was added and the tubes were properly sealed using the caps provided, shaken vigorously for 15 sec**,** centrifuged at 8000 rpm for 1 min, then incubated at 56°C for 10 min with continuous mixing. This was followed by adding 200 *μ*L ethanol (96–100%) with vigorous shaking of the tubes for 15 s. The mixture (maximum 900 *μ*L) was then carefully transferred to the DNeasy columns in the tubes which were then centrifuged for 1 min at 8000 rpm to allow passage of the lysate through the membrane of the DNeasy columns. To remove the residual contaminants, 500 *μ*L of wash buffer AW1 was added to each sample and centrifuged for 1 min at 8000 rpm, followed by 500 *μ*L of the second wash buffer AW2 added to each sample with centrifugation for 3 min at 14 000 rpm. To elute the DNA, the columns were placed in an elution tubes and a volume of 100 *μ*L AE elution buffer was added to each tube and incubated for 5 min at room temperature (15–25°C), then tubes were centrifuged for 1 min at 8000 rpm and the eluted DNA was collected and stored at −20°C for further molecular analysis.

### Molecular typing of *Brucella* species

Multiplex PCR (Bruce‐ladder) was adopted from García‐Yoldi *et al*. (2006) for the identification and differentiation of *Brucella* species. To obtain maximal band intensities for each of the *Brucella* gene amplicons, key parameters like annealing temperature, primer concentration, Mg^2+^ concentration, extension time and the amount and quality of Taq polymerase, several tests were performed to optimize the Multiplex PCR conditions (data not shown). Seven *Brucella* species‐specific primer set and DNA extracted from *three Brucella isolates* were used.

For the investigations, seven primer pairs (Fermentas, USA) were used as described by García‐Yoldi *et al*. (2006) and Lopez‐Goni *et al*. (2008) except the primer pair amplifying *Brucella suis* fragment (272 bp) was excluded because no pigs are reared in Oman. All primers were combined in one tube, and the primer mix was adjusted to a final concentration of 50 nmol/L (0.5 *μ*mol/L per primer) using 0.1X TE (10 mmol/L Tris–HCl, 0.1 mmol/L EDTA) buffer (Amresco, USA). The assay was carried out in a 25 *μ*L reaction mixture containing 12.5 *μ*L of Platinum Master Mix (Life Technologies, USA), 5 *μ*L of RNase‐Free Water (Qiagen, Germany), 5 *μ*L of primer mix and 2.5 *μ*L template DNA (0.1–0.2 *μ*g). For control reactions, template DNA was omitted and the final reaction volume was adjusted to 25 *μ*L with RNase‐Free Water.

Thermal cycling was performed with a Veriti Thermal cycler (Applied Biosystems). After initial denaturation (95°C/30 s), the PCR profile was modified from the original protocol of García‐Yoldi *et al*. (2006) and was done as follows: 40 cycles of denaturation (95°C/30 s), annealing (60°C/45 s) and extension (72°C/90 s), with a final extension step (72°C/10 min). The bands were visualized under UV light using Gel Doc XR+ system (Bio Rad, USA).The expected sizes of the amplification products for *B. melitensis* were 1682, 1072, 794, 587, 450 and 152 bp.

### Questionnaire design and data collection

Based on the serological tests results, a questionnaire has been developed to assess the farmer's daily practice with the animals to determine associated factors that might contribute to or limit the spread of the disease to different villages in Al Jabal Al Akhdar. The questionnaire contained about 42 questions covering the daily practices of the farmers with their animals, their knowledge of brucellosis, risk factors predisposing to *Brucella* infection in herds with specific questions on herd size, herd composition (presence of goat breeds other than Al Jabal Al Akhdar and other small ruminants), occurrence of abortions in the herds and the introduction of a new goat bought at the livestock market into the herd. In addition, there were some questions on risk factors predisposing to *Brucella* infection in humans, which included drinking of raw or unpasteurized milk. The types of the questions involved were yes or no questions (closed‐ended) and short‐answer questions. About 30 people, who were responsible for the daily management of the goats in the eight villages, were interviewed orally in Arabic at their households. Data were collected using paper‐based questionnaires and the data collections were completed at the selected herd sites on a single visit.

### Data analysis

The data obtained from the serological tests were entered into a Microsoft Excel spreadsheet. The total prevalence of brucellosis for individual tests was calculated by dividing the number of RBT, iELISA or CFT positive animals by the total number of animals that were tested. The true seroprevalence was estimated by dividing the number of animals tested positive in two or all three tests by the total number of animals tested. The individual level prevalence was estimated by dividing the number of seropositive animals by the total number of animals that were tested. The herd level prevalence was calculated by dividing the number of herds with at least one reactor in RBT, iELISA and/or CFT by the number of all herds tested. A Chi‐square (x^2^) test was done to compare the prevalence of brucellosis between the villages. The association between risk factors and seropositivity to *Brucella* species was considered as significant at *P* < 0.05. The comparative efficacy of RBPT, I‐ELISA and CFT was determined with regards to their overall agreement in the diagnosis of brucellosis and they were measured using Kappa statistics. Descriptive analysis was used for the questionnaire.

## Results

### Detection of *Brucella* antibodies using serological tests

#### Rose Bengal test

As shown in Table 2, all serum samples from the eight villages sampled were first screened for *Brucella* antibodies using RBT. Thirty‐eight out of 324 samples were serologically positive (11.7%). The highest serologically positive samples were recorded in animals from Al Helailat with a 40% (24/60), followed by Al Aqaieb 28% (14/50). The samples from the rest of the villages were serologically negative.

**Table 2:** Seroprvalence of *Brucella* infection in goats in Al Jabel Al Akhdar using individual serological tests (RBT, iELISA and CFT) and the true seroprevalence of animals that tested positive in RBT, iELISA and/or CFT

Village name	RBT		iELISA		CFT		*Brucella* seropositive animals	
	Positive	%	Positive	%	Positive	%	Positive	%
Al Aqayib	14/50	28.0	22/50	44.0	12/50	24.0	12/50	24.0
Al Helailat	24/60	40.0	40/60	66.7	15/60	25.0	24/60	40.0
Al Ghilayil	0/22	0.0	0/22	0.0	0/22	0.0	0/22	0.0
Al Qasha'e	0/31	0.0	0/31	0.0	0/31	0.0	0/31	0.0
Da'anAlhamra	0/41	0.0	0/41	0.0	0/41	0.0	0/41	0.0
Al Sarah	0/22	0.0	0/22	0.0	0/22	0.0	0/22	0.0
Hail Al Hedap	0/18	0.0	0/18	0.0	0/18	0.0	0/18	0.0
Shnoot	0/80	0.0	0/80	0.0	0/80	0.0	0/80	0.0
Total	38/324	11.7	62/324	19.1	27/324	8.3	36/324	11.1

RBT, Rose Bengal Test; iELISA, indirect Enzyme‐linked Immunosorbent Assay; CFT, Complement Fixation Test.

#### Indirect enzyme‐linked immunosorbant assay

All serum samples were then subjected to I‐ELISA as a confirmatory test. 19.1 per cent (62/324) of the samples were serologically positive (Table 2). The highest brucellosis seropositivity was recorded in Al Helailat with 66.7% (40/60), followed by Al Aqaieb 44% (22/50). The samples from the rest of the villages were all serologically negative.

#### Complement fixation test

For a comprehensive and comparative serological analysis, all the samples were subjected to CFT. From the results obtained (Table 2), CFT test showed the lowest percentage of seropositivity with 8.3% (27/324) compared to RBT and I‐ELISA. In line with RBT and I‐ELISA, the highest seropositivity was recorded in Al Helailat 25% (15/60), followed by Al Aqaieb with a seropositivity of 24% (12/50). No seropositive samples were recorded from the rest of the villages.

#### Overall performance of the serological tests

As illustrated in Table 2, the performance of I‐ELISA, RBT and CFT was 19.1%, 11.7% and 8.3%, respectively.

The overall seroprevalence of *Brucella* infection in goats was determined based on an animal being positive in RBT, iELISA and/or CFT due to the strong association between them and was taken for subsequent data analysis. This resulted in an overall seroprevalence of 11.1% (36/324) of *Brucella* infection in Jabel Al Akhdar.

#### Comparative analysis of the serological tests used

**Figure 1:**
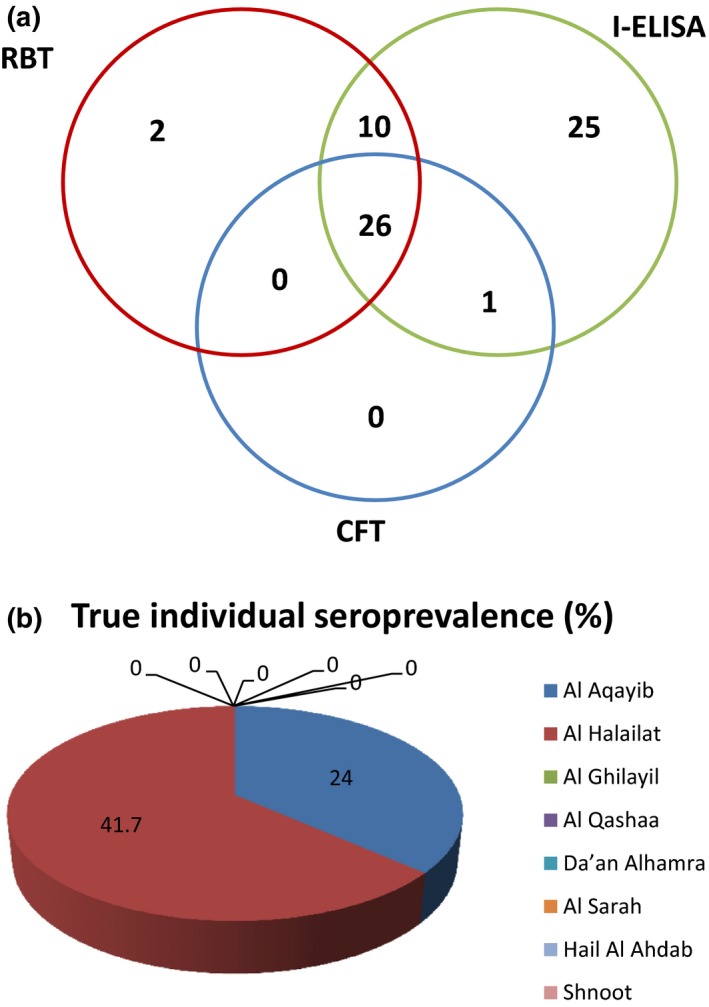
(a) Venn diagram showing a summary of serological test results (RBT, I‐ELISA and CFT) for serum samples from goats in Al Jabal Al Akhdar. As shown, 25, 2 and 1 serum samples were positive in I‐ELISA, RBT and CFT, respectively. 10 samples were positive in I‐ELISA and RBT. 26 samples were positive in all three tests. (b) The true individual seroprevalence in all eight villages.

When considering the performance of all the three serological tests together, the breakdown of the results (Table 3 and Figure 1a) was as follows: Twenty‐six samples (8.02%) tested positive in all the three tests (12 samples from Al Aqaieb and 14 samples from Al Helailat). One sample (0.30%) (From Al Helailat) tested positive with both CFT and I‐ELISA but not with RBT. Ten samples (3.08%) (All from Al Helailat) tested positive with both RBT and I‐ELISA but not with CFT. Two samples were positive only with RBT (from AlAqaieb). Twenty‐five samples (7.71%) were positive only with iELISA. Two samples (0.61%) were positive only with RBT.

**Table 3:** Comparison of the serological test results

Number of serum samples	RBT	iELISA	CFT
260	**−**	**−**	**−**
26	**+**	**+**	**+**
25	**−**	**+**	**−**
10	**+**	**+**	**−**
2	**+**	**−**	**−**
1	**−**	**+**	**+**
Total	38	62	27

The degree of agreement between RBT, I‐ELISA and CFT was then subjected to statistical analysis using Kappa statistics. An inter‐rater reliability analysis using the Kappa statistics was performed to determine agreement among these tests. As shown in Table 4, the agreement between I‐ELISA and CFT performance was moderate (*k* = 0.555 each). Kappa value for RBT and I‐ELISA performance indicated a ‘good’ agreement (*k* = 0.672). Also a good agreement between RBT and CFT performance was recorded (*k* = 0.778).

**Table 4:** Degree of agreement between different tests used in goats (*n* = 324) tested for brucellosis using Kappa statistics

Comparison	Observed Agreement	95% CI of Agreement	SE	Kappa Value	95% CI of Kappa	*P* Value	Strength
RBT Vs iELISA	91.4%	87.8–94.2	0.056	0.672	0.562–0.783	<0.01	Good
RBT Vs CFT	96.0%	93.2–97.8	0.059	0.778	0.663–0.894	<0.01	Good
CFT Vs iELISA	89.2%	85.3–92.4	0.064	0.555	0.430–0.680	<0.01	Moderate

RBT, Rose Bengal Test; iELISA, indirect Enzyme‐linked Immunosorbent Assay; CFT, Complement Fixation Test; CI, Confidence Interval; SE, Standard Error; P, Precision.

### Individual and herd‐based seroprevalence of *Brucella* infection in Al Jabal Al Akhdar

As shown in Table 5 and Figure 1b, out of the total 324 serum samples tested, the highest individual level seroprevalence of brucellosis was recorded in Al Helailat (41.7%) followed by Al Aqaieb with seroprevalence of 24.0%. No seroprevalence was recorded in animals from Al Ghilayil, Hail Al Hedap, Da'an Al Hamra, Shnoot, Al Qashaa or Al Sarah. The true individual seroprevalence of brucellosis in Jabel Al Akhdar was 11.1% (36/324) with a range 0–41.7% in the eight villages covered in this study.

**Table 5:** Individual and herd based seroprevalence of brucellosis in goats in Jabel Al Akhdar

Village name	Individual level		Herd level	
	Positive	Prevalence (%)	Positive	Prevalence (%)
AlAqaieb	12/50	24.0	3/3	100.0
Al Helailat	24/60	40.0	3/3	100.0
AlGhilayil	0/22	0.0	0/2	0.0
Al Qasha'e	0/31	0.0	0/1	0.0
Daan AlHamra	0/41	0.0	0/2	0.0
Hail Al Yamen	0/22	0.0	0/2	0.0
Hail Al Hedap	0/18	0.0	0/1	0.0
Shnoot	0/80	0.0	0/12	0.0
Total	36/324	11.1	6/26	23.1

Regarding herd‐based seroprevalence, the sampling plan included 26 herds. Only animals from six herds (23.1%) have shown evidence of exposure to *Brucella* (Al Aqayib and Al Helailat each with three herds).

The true individual seroprevalence (11.1%) was reported in Al Aqaib and Al Helailat. A Chi‐square (x^2^) analysis revealed that the individual seroprevalence of brucellosis varied significantly among the villages sampled (Chi‐square = 89.677, df = 7, *P* < 0.01). Moreover, the herd level seroprevalence was also significantly different (Chi‐square = 26.000, df = 7, *P* < 0.01).

### Seroprevalence of Brucella infection in goats, according to sex, age and abortion history

As shown in Table 6, a Chi‐square (x^2^) analysis revealed that male animals showed a slightly higher seropositivity (16%) than female animals (11%). However, the difference was not statistically significant (*P* > 0.05).

**Table 6:** Chi‐square analysis of association between potential risk factors and prevalence of brucellosis in goats at individual level in Jabal Al Akhdar

Variable	Category	Positive	Tested	Prevalence %	95% CI	*P* Value
Age	<1 year	1	16	6.3	0.2–30.2	Chi = 2.886 df = 3 *P* = 0.410
	1–3 years	20	206	9.7	6–14.6	
	3.1–5 years	14	88	15.9	9–25.2	
	>5 years	2	14	14.3	1.8–42.8	
Sex	Female	33	299	11.0	7.7–15.1	Chi = 0.562 df = 1 *P* = 0.454
	Male	4	25	16.0	4.5–36.1	
Abortion history	No	33	314	10.5	7.3–14.4	Chi = 8.332 df = 1 *P* = 0.004
	Yes	4	10	40.0	12.2–73.8	
Total		37	324	11.4	8.2–15.4	

CI, Confidence Interval; P, Precision.

In the same vein, the age‐specific parameter indicated that seroprevalence of brucellosis varied with age. The highest seroprevalence was observed in goats between 3.1 and 5 years of age (15.9%) followed by those above 5 years (14.3%), between 1 and 3 years (9.7%) and less than 1 year (6.3%) of age (*P* > 0.05).

A significantly (*P* < 0.05) higher prevalence was observed in goats with a history of abortion (40%) compared to those with no such history (10.5%).

In conclusion, apart from abortion history, seroprevalence of brucellosis was not significantly different among different age and sex groups (*P* > 0.05).

### Knowledge, attitudes and practices relating to brucellosis among farmers in Al Jabal Al Akhdar

The majority (83%) of the respondents were selling their goats on a regular basis with mostly 1–5 heads sold per year, mainly in the Nizwa local market. Eighty‐three per cent of the respondents were not buying new goats. All respondents were milking their goats, with the majority (93%) milking them twice a day. Seventy‐three per cent of the respondents were boiling the milk for 5–10 min before drinking it to kill germs as they stated. Twenty‐seven per cent of the respondents were not heating the milk for the purpose of making Laban (sour milk). Respondents from Al Helailat and Al Aqaieb were among those who are making Laban on a daily basis. None of the respondents were selling the milk to other people in or outside the village. All respondents stated that their goats had had an abortion during the last year with 57% of owners suffering 10–20 abortions in their herd per year. All respondents allowed their goats to graze freely during the day and some of the herds became mixed with other farmers' herds. Goats from Al Helailat were mixed with other goats all day, whereas respondents from Al Aqaieb did not allow their goats to mix with other goats. Seventy‐three per cent of the respondents have the Al Jabal Al Akhdar breed only in their herds, whereas 27% (including herds from Al Aqaieb and Al Helailat) had mixed breeds of Batina and Dhofari. None of the respondents knew the vaccination history of their goats. Seventy per cent of the respondents did not keep goats with sheep, whereas 30% mixed them together (including farmers from Al Helailat). All the respondents stated that they washed their hands each time they handled any animal. All the respondents dealt with an aborted animal and dead foetus and the majority (97%) took specific actions like wearing gloves and washing hands. Only one respondent (3%) did not take specific precautions. All the respondents dealt with a dead foetus by throwing it away. All the respondents took immediate action by quarantining when they suspected an animal was sick, and sought veterinary help. Ninety per cent of the respondents quarantined any new animals bought before introducing them to the herd and about 10% did not take any action when buying a new animal and immediately introduced it to the herd.

### Farmers' knowledge of brucellosis

Seventy per cent of the respondents had never heard of brucellosis. Of those who had heard of the disease (*n* = 9), the majority (*n* = 6) had received information via lectures conducted by the Ministry of Agriculture and Fisheries (MAF), but only 44% knew that cattle, sheep or goats could become infected. All respondents who had heard of brucellosis knew that humans could become infected through drinking raw milk and responded that arthritis was a common symptom in humans. Fewer (*n* = 4) knew one correct route of transmission between animals. The majority of the respondents, who had heard of brucellosis (88%), assumed that any family member could become infected with brucellosis at any age. People in Al Aqaieb and Al Helailat who have heard of brucellosis, stated that 6 and 5 family members, respectively, had been diagnosed with the disease by a physician.

### Identification of *Brucella melitensis* from bacterial isolates

Although 37 samples showed strong seropositivity in serological tests, only three (8.1%) bacterial isolates were obtained from the culture. Two isolates were obtained from milk sample cultures and one isolate was obtained from a blood sample culture. Using serum agglutination test, the bacterial isolates were initially identified as *Brucella melitensis*. Gram staining of the bacterial isolates showed a typical morphology for Gram‐negative *coccobacilli* bacteria (data not shown). Moreover, the three isolates gave 99% identification probability for *Brucella melitensis* when entered into Vitek 2 system (Gram‐negative card).

### Multiplex PCR

**Figure 2:**
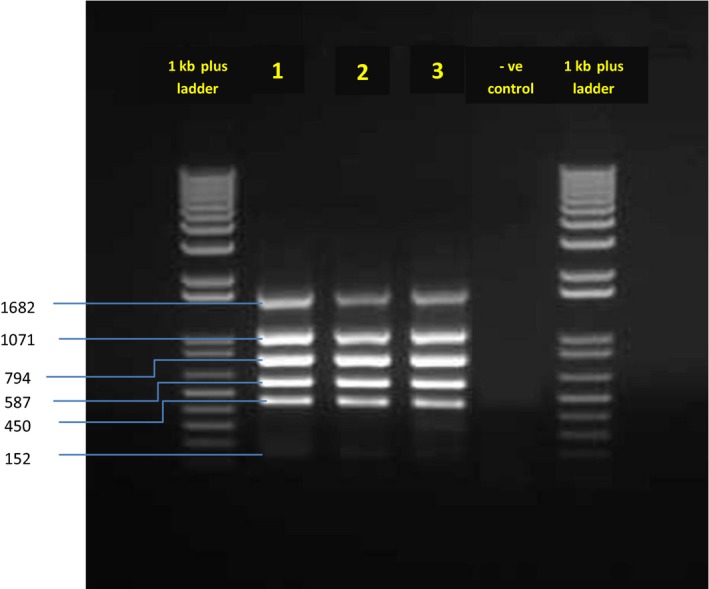
Agarose gel electrophoresis of species‐specific Multiplex PCR products. Lane 1 and 6 contain 1 kb plus ladder. Lane 2, 3 and 4 contain different *brucella* isolates. Lane 5 contains negative PCR control.

As shown in Figure 2, Multiplex PCR electrophoresis illustrated the presence of six amplified bands, of 1,682, 1,071,794, 587, 450 and 152 bp in size, a typical profile for B. melitensis. These results confirmed the specificity and sensitivity of these primers for the targeted regions in Brucella DNA.

## Discussion

The objectives of this study were primarily to investigate the seroprevalence of brucellosis, associated risk factors and identification of *Brucella* species among goats in Al Jabal Al Akhdar, Sultanate of Oman, aiming for quick and accurate diagnosis of brucellosis, which is very important for a positive outcome of eradication and monitoring programs. In this study, three different serological tests were applied and their performance was evaluated. All 324 serum samples within the study area were tested for *Brucella* antibodies using RBT, I‐ELISA and CFT independently. The performance of I‐ELISA, RBT and CFT was 19.1%, 11.7% and 8.3%, respectively. The overall seroprevalence of *Brucella* infection in goats was determined based on an animal being positive in RBT, iELISA and/or CFT, which resulted in an overall seroprevalence of 11.1%. In this study, 25 samples tested positive by iELISA, but negative by both RBT and CFT. This could be explained by either: (i) higher sensitivity of iELISA because it uses cytosolic S‐LPS fragments, thus decreasing the cross‐reaction with other Gram‐negative bacteria (Araj *et al*. 1986; Jacques *et al*. 1998; Nielsen 2002; Corbel 2006; Office International of Epizooties, 2012), (ii) prozoning phenomena that occurs usually in acidified antigens in RBT, (iii) anti‐complementary activity in CFT (Nielsen 2002). On the other hand, two samples tested weak positive by RBT, but negative by both iELISA and CFT. This might be a result of cross‐reacting antibodies produced due to infection with other Gram‐negative bacteria as three other samples that tested as a weak positive with RBT, also tested positive with iELISA. Moreover, only one sample tested positive by iELISA and CFT but negative by the RBT. This could be attributed to the use of standardized RBT method that implies the use 1:1 ratio of the serum and the antigen, which is not the optimum ratio to use for goat sera (3:1). This might lead to decreased sensitivity of the RBT and therefore, missed the detection of antibodies in that animal serum. Ten sera tested positive by both RBT and iELISA, but negative by CFT. This could be explained by the anti‐complementary activity due to inactivation or destruction of the guinea pig complement by the serum that resulted in a false‐negative result. This finding agreed with a study performed by Blasco *et al*. (1994) to test the efficacy of different RBT and CFT antigens for the diagnosis of *Brucella melitensis* infection in sheep and goats. They concluded that the CFT test was less sensitive but more specific than RBT test. ELISA has been evaluated for many years for their better sensitivity to detect anti‐*Brucella* antibodies in all species. Several studies reported that iELISA is more sensitive than conventional tests (Nielsen 2002). Jacques *et al*. (1998) assessed the efficacy of indirect ELISA in comparison with RBT and CFT on sera from ewes infected with *Brucella melitensis*. The indirect ELISA was shown to be a good screening test and could be used alone or in addition to RBT. The present I‐ELISA performance is also in agreement with Nielsen *et al*. (2004), as they compared different serological tests with experimentally vaccinated and infected group of sheep and goats. They concluded that I‐ELISA outperformed RBT and CFT and recommended it for the diagnosis of Ovine‐Caprine brucellosis, though RBT performance in this study contradicts their outcome. This might be explained by the fact that the authors used experimentally infected and vaccinated animals, which is not the case in this study where there is no vaccination history in the animals tested that would contribute to a false positive outcome. Thus serological tests should be analysed according to the true infectious status of an animal (Bevins *et al*. 1996) as the presence of anti‐*Brucellae* antibodies may not mean that the animals have a current or active infection at the time of sample collection.

An inter‐rater reliability analysis using the Kappa statistics showed that the degree of agreement was best seen between RBT/CFT (96%) followed by RBT/I‐ ELISA 91.4%, and CFT/I‐ ELISA 89.2%. The strong association between the RBT and CFT could be explained by the fact both RBT and the CFT use whole‐cell antigens of *Brucella* compared with the iELISA which uses cytosolic S‐LPS fragments (Jacques *et al*. 1998; Coelho *et al*. 2013). However, results of this study contradict the results of a study conducted by Delgado *et al*. (1995) on the evaluation of ELISA for the detection of sheep infected and vaccinated with *Brucella melitensis*. They showed an excellent agreement between ELISA and the CFT (*k* = 0.89) followed by a good agreement between RBT and ELISA (*k* = 0.73).

The agreement between two tests has been suggested as an evaluation criterion for a diagnostic test (Martin 1977). The kappa measures the range of agreement between two tests and ranges from −1 to 1, where 1 is a perfect agreement, 0 is exactly what would be expected by chance and −1 is a perfect disagreement (Landis & Koch 1977; Viera & Garrett 2005).

Our results are in agreement with the recommendations of the world organization of animal health **(**2012) to use RBT and CFT as a screening and confirmatory tests, respectively; however, CFT is a complex method to perform requiring good laboratory facilities and trained staff (Corbel 2006). Therefore, this study suggests that more validation for CFT using naturally infected goats is needed.

Our results indicate that it is satisfactory to use RBT/I‐ELISA as screening and confirmatory tests, respectively, based on a good agreement between them. These results highlight the importance of using more than one type of diagnostic technique for the detection of animals positive for brucellosis, especially for epidemiological purposes.

The gold standard in brucellosis diagnosis remains the isolation of *Brucella organisms*. Though there are many reports on isolation of *Brucella* from milk samples (Leal‐Klevezas *et al*. 1995;  Hamdy & Amin 2002), few studies are based on detection of *Brucella* from blood samples (Leal‐Klevezas *et al*. 1995).

In this study, no isolates of *Brucella* were recovered from milk or blood samples of goats using Farrell's modified serum dextrose agar (data not shown) which has been previously used successfully to isolate *Brucella spp*. There are a number of possible reasons for the failure of culture. This could be due to the intermittent excretion of organisms in milk, no bacteraemia, too few bacteria in the sample or due to a low volume of milk inoculated (Alton & Paris‐Grignon 1988). Zambriski *et al*. (2012) showed that culture from fresh samples or samples stored at refrigeration temperature is normally easy. In the case of blood culture, reports indicate that low isolation rates could be attributed to that bacteria lacked sufficient sensitivity (Gupta *et al*. 2006).

To overcome these problems, fresh milk and blood samples from serologically strongly positive animals were inoculated into BACTEC bottles and incubated for about 7 days in a BACTEC 9240 system. Samples from positive bottles were subcultured in 5% sheep blood agar and incubated for 3–4 days at 37°C. Subcultures from negative bottles were considered negative if no *Brucella* was detected during the 4 weeks incubation. This procedure yielded three isolates (two from milk and one from blood samples). All gram staining and biochemical tests revealed a typical profile of *Brucella melitensis*. As this was a protracted procedure, only 10 samples were tested. Therefore, culture performance could not statistically be compared to the performance of the serological tests.

The use of Bruce‐ladder multiplex PCR allowed us to detect, for the first time, *B. melitensis* in goats in Al Jabal Al Akhdar. Multiplex PCR using DNA from *three isolates* amplified six fragments of 1682, 1 071 794, 587, 450 and 152 bp in size, a typical profile for *B. melitensis*. The absence of the 218‐bp fragment specific for *B. melitensis* Rev.1 vaccine strain is a further proof that the animals in Al Jabal Al Akhdar were not vaccinated. Previously, Bruce‐ladder PCR has been tested (García‐Yoldi *et al*. 2006) and it demonstrated agreement of results of seven laboratories for *Brucella* samples from human as well as domestic and wild animals from five continents (Lopez‐Goni *et al*. 2008) demonstrating without a doubt the reproducibility and robustness of the PCR.

However, in this study, this PCR gave confusing bands when DNA from milk or blood samples was used directly as a template. Moreover, this PCR assay cannot differentiate biovars from the same species**.** It may be that further classical typing and multiple‐locus variable‐number tandem‐repeat analysis is needed to identify these isolates to their biovar level.

This study also demonstrates an unevenly distributed seroprevalence among different villages located within of 1.53 km and 19.28 km from the disease hot spots villages of Al Helailat (41.7%) and Al Aqaieb (24%). It is possible that certain risk factors are more widespread in some villages. However, it should be noted that serologically negative animals may be incubating the disease and present a risk (Corbel 2006), which necessitates constant testing. Geographical variation in goat brucellosis prevalence between certain communities was reported in Ethiopia (Tschopp *et al*. 2015) as the authors explained that pastoralists tend to trade animals with pastoralists from their own ethnicity/clan rather than with neighbouring pastoralists of other ethnic groups. This study found trade and husbandry systems could be major risk factors which certainly need consideration in Al Jabal Akhdar communities which is in agreement of observations elsewhere (Jackson *et al*. 2014, Ward *et al*. 2012).

Moreover, prevalence in male goats was slightly higher than in females. This could be explained by the fact that there are few bucks serving a large number of females. Similar prevalence was reported in Pakistan (Ghani *et al*. 1995), Bangladesh and Mexico (Solorio‐Rivera *et al*. 2007; Islam *et al*. 2012).

This study also compared different age groups. The lowest seroprevalence was observed in goats less than 1 year of age (6.3%), followed by goats between 1 and 3 years (9.7%), above 5 years (14.3%), 3.1 to 5  years of age (15.9%). However, there was no significant association between *Brucella* infection and age of animals (*P* > 0.0.05). These results are in agreement with other reports which showed that ovine and caprine older than 24 months of age were more likely to get *Brucella* infection than animals younger than 12 months of age. The possible explanation is that older animals could have greater chances of exposure to infected herds or animals (Tsegay *et al*. 2015; Ashagrie *et al*. 2011). However, further studies need to be conducted to explore this aspect.

Numerous factors are involved in the epidemiology of brucellosis (Food And Agriculture Organization 2010), including reproductive disorders in goats (Corbel 2006). In this study, all pastoralists (100%) stated that their goats had aborted in the year before conducting the questionnaire, with over half of them (57%) having experienced multiple abortions per year. Yet drinking raw milk is very common in the Sultanate of Oman.

Al Jabal Al Akdhar has its own unique goat breed carrying its name, but 27% of the respondents had mixed breeds of Batina and Dhofari. Introduction of breeds from endemic areas of Dhofar (Al‐Rawahi 2015) and from Albatina with recent outbreak (the Ministry of Agriculture and Fisheries, 2016) could pose a risk to the animals in the study area. Differences in goat breeds regarding brucellosis susceptibility has been reported in different countries (Solorio‐Rivera *et al*. 2007;  Ali *et al*. 2015). However, more studies are needed to correlate brucellosis and different breeds of goats in the Sultanate of Oman.

Al Jabal Al Akhdar Mountain supports vegetation growth as well as attracting visitors, which would contribute to infection and zoonoses as climatic factors naturally influence infection rates when considering human and animal hosts dynamics (Zhang *et al*. 2010).

Finally, management practices and husbandry in the villages studied were highly homogenous. Vaccination is the most effective method for prevention. To reduce the prevalence of infection, widespread mass vaccination of whole herds can be recommended wherever the disease is endemic. However, education of the population regarding brucellosis, including risk factors concerning transmission, could be useful to reduce the impact of the disease (Corbel 2006).

## Conclusions and recommendations

In conclusion, this study is the first to reveal that brucellosis is prevalent in goats in Al Jabal Al Akhdar, Sultanate of Oman with an overall prevalence of 11.1%.

This study also compared the performance of three different serological tests RBT, I‐ELISA and CFT. Statistical analysis using Kappa statistics showed the degree of agreement was best seen between RBT and CFT (96%) followed by RBT and I‐ ELISA 91.4%, and CFT and I‐ ELISA 89.2%.

A structured questionnaire and Chi‐square (x^2^) statistical analysis identified that presence of abortion is the major risk factor for brucellosis, whereas age and sex played no significant role in the animals tested. Besides, poor knowledge about brucellosis, consumption of unpasteurized milk or milk products, free trade and the introduction of new animals to herds without quarantine are all potential contributing risk factors to the prevalence of brucellosis. The prevalence of human brucellosis obtained verbally from pastoralists gave an insight that brucellosis could pose a significant public health hazard, especially in high‐risk groups, mainly pastoralists. Because of their constant and increased interaction with their animals, pastoralists could be at a high risk of occupational infection. Based on the present findings the following recommendations are worth mentioning:

1. A combination of RBT, iELISA and/or CFT is needed, to test the samples in parallel, to detect *Brucella* infection in the Sultanate of Oman.
2. Further validation for CFT using naturally infected goats is needed.
3. Further studies are needed to employ better antigens for the detection of anti‐*Brucella* antibodies.
4. Development of an immunochromatographic assay that is rapid, non‐expensive, economical and suitable for large‐scale screening in rural areas and could be integrated into other diagnostic procedures.
5. More studies are needed to correlate brucellosis with different breeds in the Sultanate of Oman.
6. Pastoralists need to be educated on the public health hazard of *Brucella* infection.
7. Restriction of animal movement and trade need to be implemented by the authorities.
8. There is an immense need to develop a control programme that includes vaccination, screening and culling of goats showing serological evidence of *Brucella* infection in the study area.

## Source of funding

This work was supported by grant from HIS MAJESTY's Sultan Qaboos Bin Said for strategic research fund (project no. SR/ANVS/14/01).

## Conflict of interest

All the authors declare no conflict of interest in this paper.

## Ethics statement

In this study, verbal consent of farm owners was obtained prior to the collection of blood samples from their animals. Animals were used just once for jugular venipuncture by professional veterinary technologists at the Department of Animal & Veterinary Sciences. This work was for diagnostic purposes and not an experiment and hence approval by the ethical committee at Sultan Qaboos University was not needed.

## Contributions

Design of the experiments: Yasmin ElTahir & Eugene H. Johnson; Samples collection and conduction of experiments: Al Ghalya Al Toobi, Osman Mahgoub, Maryne Jay, Yannick Corde, Hadi Al Lawati, Abeer Al Hamrashdi, Kaadhia Al Kharousi, Nasseb Al‐Saqri, Rudaina Al Busaidi, Ehsan Mekki, Umaima Al Hanai and Sunil Rajamony; Statistical analysis: Shekar Bose; Manuscript draft and Revison: Yasmin ElTahir, Waleed Al‐Marzooqi, Osman Mahgoub, & Eugene H. Johnson.

## Supporting information


**Appendix S1.** Impacts.

**Map S1.** The map of the geographical locations of the villages studied on Jabal Al Akhdar.
