# Supplementary material for: Serological, cultural and molecular evidence of Brucella melitensis infection in goats in Al Jabal Al Akhdar, Sultanate of Oman
Source: Vet Med Sci. 2018 May 23;4(3):190–205. doi: 10.1002/vms3.103 (PMC6090411; doi:10.1002/vms3.103)
Supplement: Supplementary file 1 — Appendix S1. Impacts. [file VMS3-4-190-s001.docx]

**Impacts**

- The present study is the first to reveal that brucellosis is prevalent in goats in Al Jabal Al Akhdar, Sultanate of Oman with an overall prevalence of 11.1%.
- Statistical analysis using Kappa statistics showed the degree of agreement between the performances of three different serological tests was best seen between RBT and CFT (96%) followed by RBT and I- ELISA 91.4 %, and CFT and I- ELISA 89.2%.
- The use of Bruce-ladder multiplex PCR allowed us to detect, for the first time, *B. melitensis* in goats in Al Jabal Al Akhdar.
