# Supplementary material for: Serological, cultural and molecular evidence of Brucella melitensis infection in goats in Al Jabal Al Akhdar, Sultanate of Oman
Source: Vet Med Sci. 2018 May 23;4(3):190–205. doi: 10.1002/vms3.103 (PMC6090411; doi:10.1002/vms3.103)

The map of the geographical locations of the villages studied on Jabal Al Akhdar

Source of Al Jabel Al Akhdar map: Esri, DigitalGlobe, GeoEye, Earthstar Geographics, CNES/Airbus DS, USDA, AEX, Getmapping, Aerogrid, IGN, IGP, swisstopo, and the GIS User Community.

Source of Oman map: Esri, HERE, DeLorme, TomTom, Intermap, increment P Corp., GEBCO, USGS, FAO, NPS, NRCAN, GeoBase, IGN, Kadaster NL, Ordnance Survey, Esri Japan, METI, Esri China (Hong Kong), swisstopo, MapmyIndia, ©OenStreetMap contributors, and the GIS User Community.

Both maps have been created using a program called ArcGIS 10.2

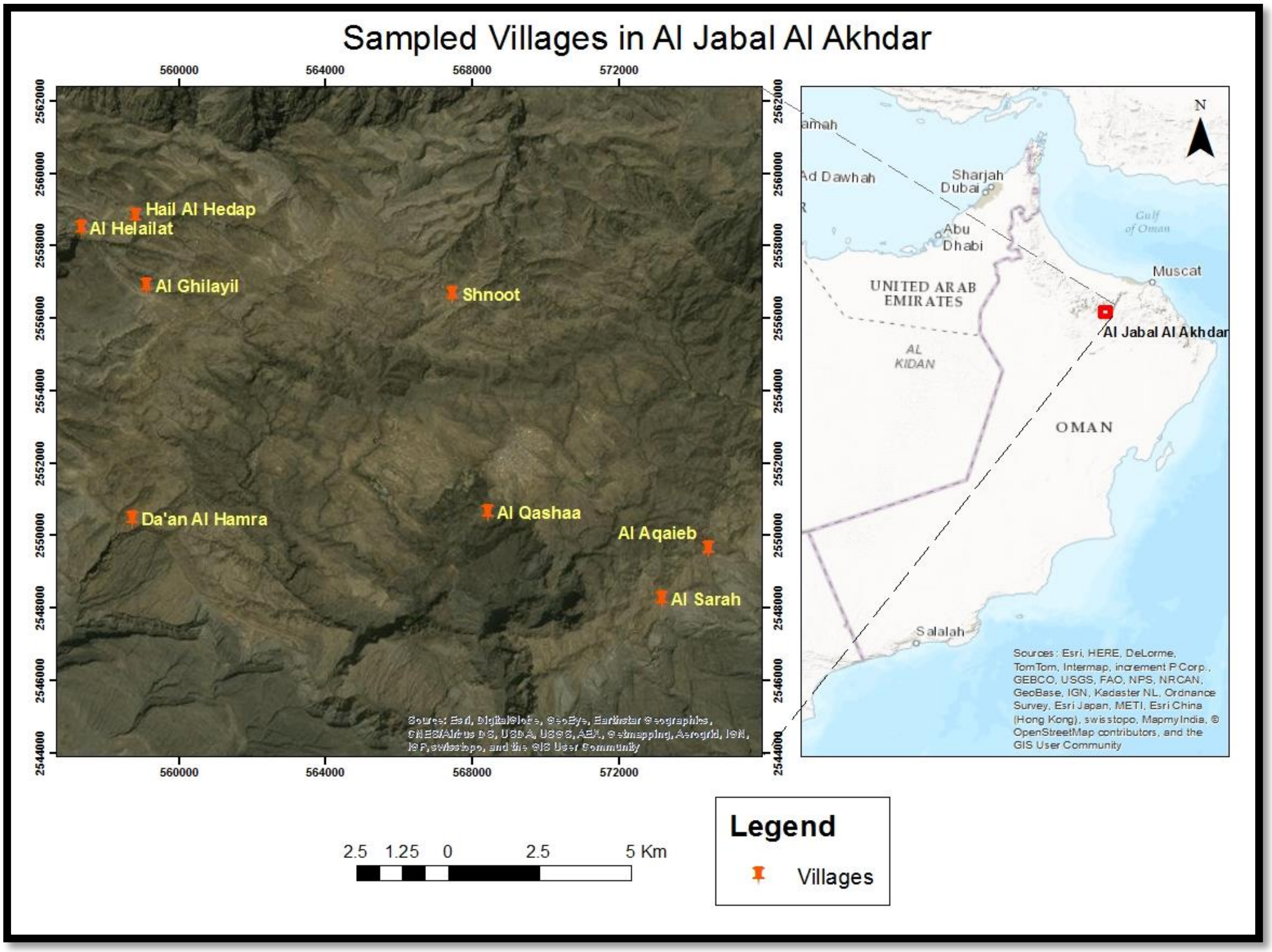

Supplement: Supplementary file 2 — Map S1. The map of the geographical locations of the villages studied on Jabal Al Akhdar. [file VMS3-4-190-s002.pdf]
